# Influence of the pressure of compacted glass powders on the final structure of sintered glass-ceramics

**DOI:** 10.1016/j.heliyon.2024.e39237

**Published:** 2024-10-11

**Authors:** N.B. Jordanov, D. Tatchev, E. Karamanova, A. Karamanov

**Affiliations:** Institute for Physical Chemistry, Bulgarian Academy of Sciences, Bl. 11, Acad. G. Bonchev Str., 1113, Sofia, Bulgaria

**Keywords:** Optical dilatometry, Press powders, Glass, Sintering, Crystallization, Porosity

## Abstract

In reported investigation we studied sintered glass-ceramics from a model diopside glass with moderate crystallization ability by *in-situ* optical dilatometeric experiments. The initial glass was ground and sieved below 75 μm. Then thus prepared powders were pressed with different pressures between 50 and 250 MPa. In this way were obtained samples out of pressed powders with different initial porosities determined with gas pycnometry. Then the samples were heat treated in a contactless horizontal optical dilatometer and their porosities were measured one more time. Both pressed green samples and synthesized glass-ceramics were analyzed further with X-Ray micro tomography.

We observed that the densification of all samples completes in the same moment which corresponds to the formation of a critical percentage crystal phase. Notwithstanding the continuation of the crystallization process, we obtain materials with different percentage of residual porosity. Then no volume variation of the system is observed. This approach is well demonstrated by the first derivative of the shrinkage curves with time.

After the end of the shrinkage of the system, the crystallization leads to the formation of crystallization induced porosity (called by us CIPs) observed here.

## 1 Introduction

Sinter crystallization is an original method towards the synthesis of new glass-ceramic materials, combining the ability of a ground glass frit to densify and the ability of the glass to crystallize to a certain extent [1,2]. Both processes are competing with each other, thus requiring a controlled heat treatment by means of programming heating rates, holding times and temperatures of exposure [[3], [4], [5], [6], [7], [8], [9]].

Obtained from an appropriate parent glass by heat treatment, the sintered glass ceramics usually contain several types of pores [10,11]. The most studied one is the residual intergranular porosity. Sometimes also are present additionally intragranular pores which are by nature of origin named crystallizationally induced pores (CIPs). Latter are being formed due to the present density differences between the amorphous and the crystalline state [10].

The firing regime depends on different factors: e.g. granular composition, size distribution, viscosity and the pressing force as well. The higher the particle size or viscosity, the longer is the sintering time at a fixed temperature. Other important factor is the initial porosity which mainly depends on the applied forming pressure. In current investigation we study the influence of this pressing force on final structure.

At the higher initial porosity of the green samples bigger shrinkage and longer sintering times are expected. However, in the case of sintered glass-ceramics, sometimes the sintering times are limited by the conquering crystallization process.

In this case variations in the pressing force of the ground initial glass frit could influence the final degree of sintering of the newly obtained glass-ceramics and the pore populations distributions in the bulk.

Besides the research of Bethanis et al. [12] we aim to investigate the influence of green strength on the both conquering processes sintering and crystallization by means of optical dilatometry.

This represents in fact the motivation of current research: to prepare initial green samples by pressing with different applied pressures (e.g. at 50 and 200 MPa) and to study *in-situ* in a contactless horizontal optical dilatometer (ODLT) instrument, the sintering curves obtained during heat treatment of standard samples.

## 2 Experimental procedure

### 2.1 Materials

We have used in current study a model laboratory diopside glass (GB-19) with the following theoretical glass batch composition in [% wt.]:

SiO_2_: 57.9; CaO: 19.3; MgO: 13.7; B_2_O_3_: 5.0; Na_2_O: 4.4;

The used chemicals in the form of oxides are Merck and Aldrich, with synthesis grade purity. Since the glass contains equimolar quantities of CaO and MgO amounting both to 19 % mol it is abbreviated as GB-19. Similar glasses have been used for many years by the working group [13].

We obtain 200 g frit after melting in a corundum crucible with an electric furnace at 1500 °C and subsequent water quenching.

### 2.2 Preparation procedure

The obtained after water quenching glass frit is further crushed, ground in a planetary mill FRITSCH (Germany) and sieved below 75 μm with a digitally programmed sieving machine CISA (Spain). The press powders are then initially humidified up to 10 % by adding aqueous solution of 7.5 % wt. polyvinyl alcohol (PVA) as a pre-pressing binding agent. PVA has no particular impact on sintering, but it is used to initially form the green sample compacts by cold pressing. The binding agent is then being burned out in a programmed isothermal stage at 270 °C inside the optical dilatometer.

### 2.3 Characterization

Samples of identical size are then mechanically homogenized and placed in a matrix with a length of 50 mm and a thickness of 5 mm in size. Then the green samples are pressed consequently in a manual hydraulic press at 50, 100, 150, 200 and 250 MPa respectively. The apparent density, *ρ*_*AP*_, of the samples is measured with a precise manual caliper and digital balances the way like this: All the three side sizes (thus obtaining the volume) are measured and the weight of the parallelepiped objects as well. The same procedure is being repeated after sintering.

Differential thermal analysis was performed with a PerkinElmer Diamond analyzer.

The sintering was carried out with constant linear heating rate of 10 °C min^−1^ with a thermal-optical measuring and imaging system ODLT-1400 MISURA ExpertLabService (Italy).

The existing crystal phases in the glass-ceramics are established by X-ray diffraction spectroscopy (XRD) with a PANALYTICAL EMPYREAN (USA) spectrometer.

The powdered samples were subject to single particle size determination in dry air by means of Mie interferometry spectroscopic analysis with a Mastersizer 3000 MALVERN (UK) instrument.

Scanning electron microscopy (SEM) images of surfaces and fractures of the sintered

glass-ceramics were taken with a JEOL 6390 (Japan) instrument with the following working parameters: working distance of 10 mm; 20 kV electron beam from a tungsten cathode.

The open porosity is determined here with Equation (1) by the product of water permeability value (obtained by soaking of the sample in boiling water for 3 h) and the apparent density after sintering, *ρ*_*AP*_ [g cm^−3^]:

(1) $$P_{O}=\rho_{AP}W\;\left[\%\right]$$

The closed porosity, *P*_*C*_ [%] is estimated as the difference between the absolute density, *ρ*_*A*_ [g cm^−3^] and the skeleton density, *ρ*_*S*_ [g cm^−3^].

(2) $$P_{C}=\frac{\rho_{A}-\rho_{S}}{\rho_{A}}100\;\left[\%\right]$$

Pycnometric determinations of densities were performed with a MICROMERITICS AccuPyc 1330 (USA) gas pycnometer (GP). This is an automated instrument, working with Helium or Argon. It allows density measurements of powders or bulk samples with arbitrary shape with a maximal resolution of 0.0003 g cm^−3^.

To obtain the absolute density of the parent glass and of the glass-ceramics, the green samples are measured as prepared in the GP. The absolute density of the obtained glass-ceramics is measured with GP as well in the end after grinding below 63 μm. Absolute density value thus obtained is 2.904 g cm^−3^ for the glass-ceramics and 2.656 g cm^−3^ for the parent glass.

In addition the porosity was evaluated numerically by 3-D computed micro-tomography (μCT). 3-D μCT was used for entire bulk scanning of the foam glass-ceramic species. The tomographic measurements were carried out with an X-ray micro-tomograph Bruker SKYSCAN 1272 (Germany), which uses a white beam with cone geometry. The following setup conditions were applied: X-ray tube voltage 70 kV, current 142 mA and 0.11 mm Cu filter. The voxel (3-D pixel) size was 1 μm and the optical magnification was 7.4. A typical 360° scan took 21 h and 27 min. Reconstruction of the 3-D images was done with the commercial software InstaRecon.

## 3 Results and discussion

**Fig. 1:**
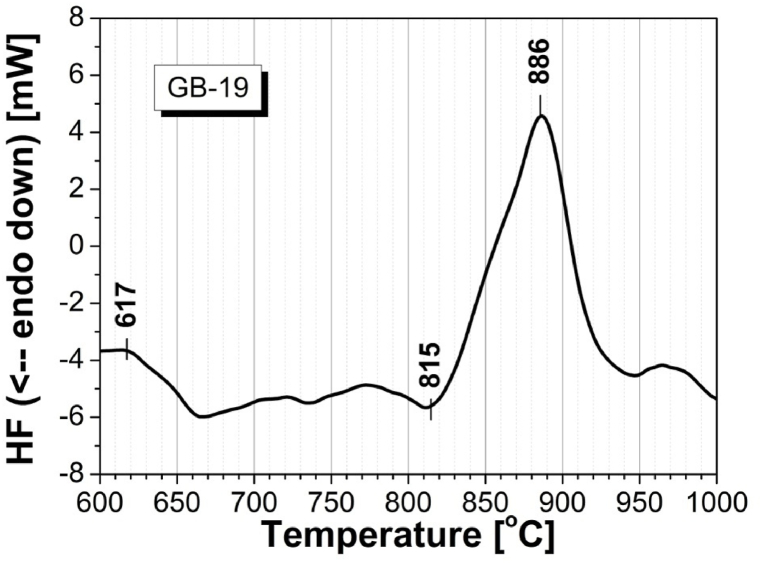
DTA curve of the process of crystallization of model glass GB-19.

**Fig. 2:**
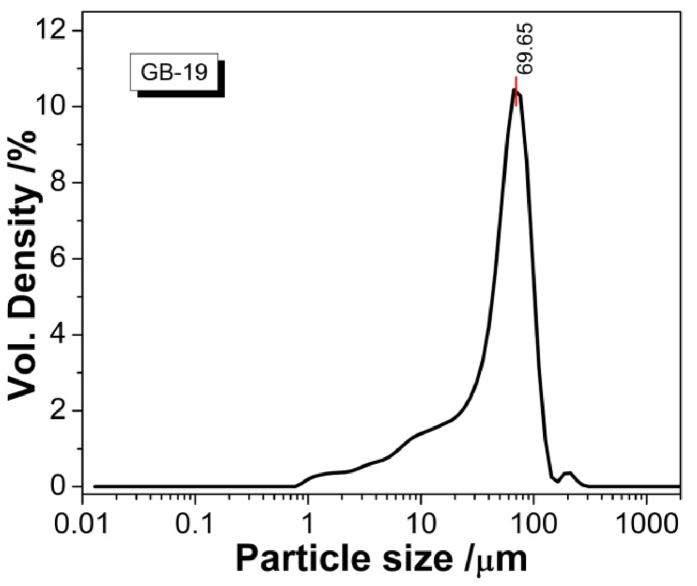
Size distribution of the particles in the sample glass GB-19.

**Fig. 3:**
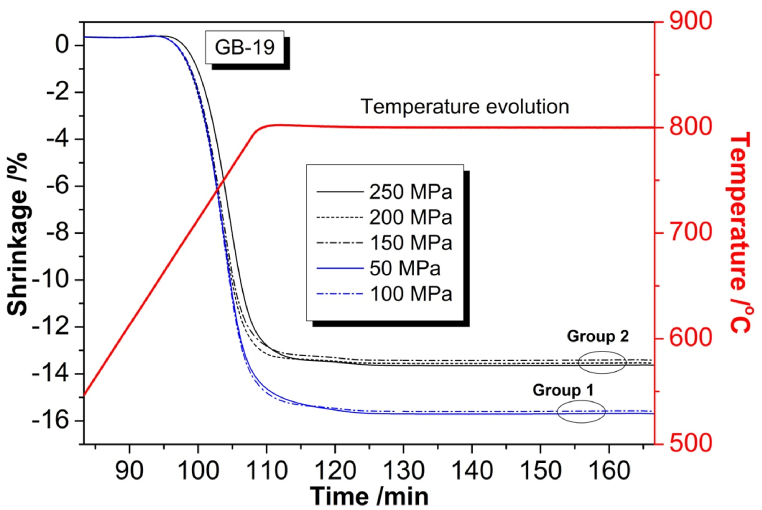
Experimental ODLT sintering curves of press powders obtained with pressures between 50 and 250 MPa.

In Fig. 1 is presented an experimental curve recorded with a differential thermal analyzer (DTA) of the crystallization process of the used model glass. At 617 °C is occurring the glass transition temperature, *T*_*g*_; The onset of crystallization is to be observed at 815 °C and the maximal crystallization peak is reached at 886 °C. This is the analysis giving the selected value for isothermal heat treatment in the ODLT instrument amounting to programmed 800 °C with holding time of 1h, as it is obvious in Fig. 3. In Fig. 2, Fig. 3 is presented a graphical representation of the size distribution of dry particles of the ground and fractionized by sieving used model glass sample GB-19. The experimental curve is uniform mono-modal with a maximum peak at approx. 70 μm.

In Fig. 3 are presented experimental sintering curves of press powders prepared with a pressure between 50 and 250 MPa, respectively. The ODLT curves in Fig. 3 are naturally grouped in two stacks by applied pressing force. It is obvious that the curves in group 1 (i.e. with lower pressing force) have reached higher shrinkage, compared to the curves in group 2 (i.e. with higher pressing force) due to higher initial porosity. However, this is not an indication by any means that higher degree of shrinkage means straight better sintering. Observing the experimental curves, we can summarize that there is of no practical significance the need to compress the green samples with pressures higher than 100 MPa, since the obtained degree of sintering is insufficient and similar with the higher applied pressures, as it is obvious from analyzing the two stacks of curves.

**Fig. 4:**
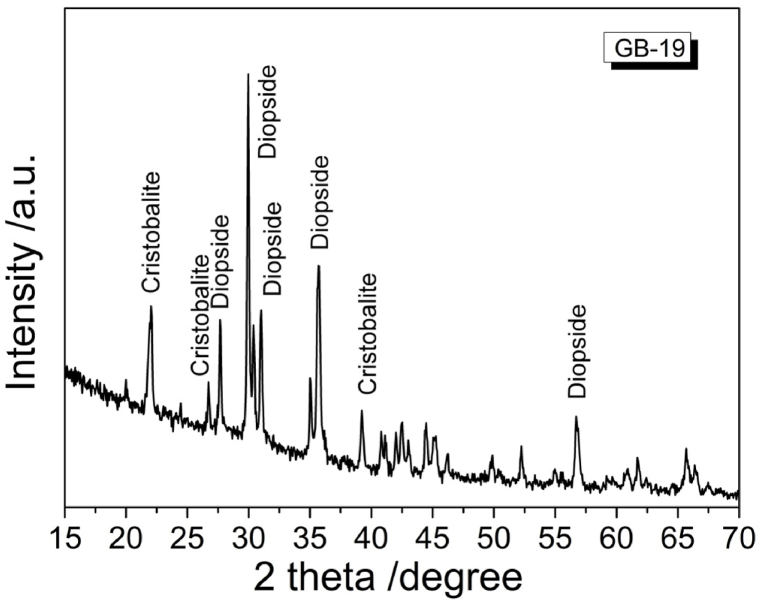
XRD spectrum of model glass GB-19 after thermal treatment.

The formed critical crystal phase in the newly obtained glass-ceramics is mainly diopside crystals phase and some cristobalite crystals as it is seen in Fig. 4. The reference pattern ICSD reference numbers for the identified phases are 98-001-0222 for diopside and 98-016-2617 for cristobalite, respectively. The pattern indicates high crystallinity which may inhibit further sintering. In this manner there is present a limited time available for sintering and a residual porosity is present.

The crystallinity of the heat treated model glass used here amounts to 55–60 % wt., which has been determined by density measurements [14].

**Fig. 5:**
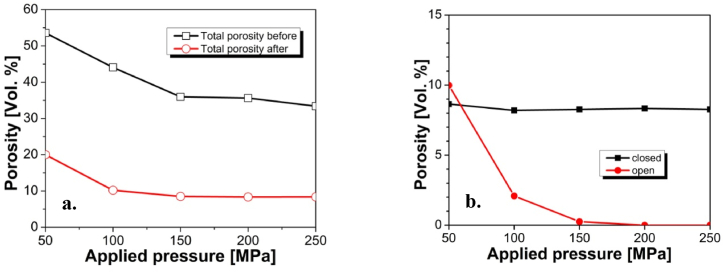
**a.** Total porosity of the initial and final sample; **b.** Open and closed porosities of the final samples.

In Fig. 5a are shown the total porosities of the green samples and of those after thermal treatment. In Fig. 5b are presented determinations of the open and closed porosities in the final heat treated samples respectively determined by water absorption and GP.

In Table 1 are listed GP values of densities measured for all pressed samples. The most deviating value is that of the 50 MPa sample pressed with the lowest load.

**Table 1:** Densities, *ρ*_*AP*_, of pressed powders, determined by GP [g cm^−3^].

Sample pressed with [MPa]:	Density:
50	**1.997**
100	**2.565**
150	**2.609**
200	**2.616**
250	**2.675**

In Table 2 are summarized data on the water permeability of the glass-ceramics obtained gravimetrically with digital balances measured before and after boiling water saturation.

**Table 2:** Water permeability of sintered pressed powders [%].

Sample pressed with [MРa]:	Water permeability:
50	**5.0**
100	**0.8**
150	**0.1**
200	**0.0**
250	**0.0**

It is evident that samples formed with lower pressure naturally possess higher porosity notwithstanding that after thermal treatment the samples shrink to a bigger extent (i.e. larger shrinkage) of about 2 % compared to higher pressed samples.

We may assume that the time available for densification is equal and the same for all samples and is further limited by the crystallization process. The samples pressed with 150, 200 and 250 MPa, respectively densify entirely before the inhibiting crystallization. These samples reach similar percentage of 14 % shrinkage (without the formation of open porosity). Since they possess similar initial porosities the reached extent of shrinkage is also similar. In the same moment, the time of sintering of the 50 and 100 MPa samples is insufficient and the samples remain with the same open porosity. This means that the theoretical sintering of the 50 and 100 MPa samples would require longer sintering times which are not available here.

**Fig. 6:**
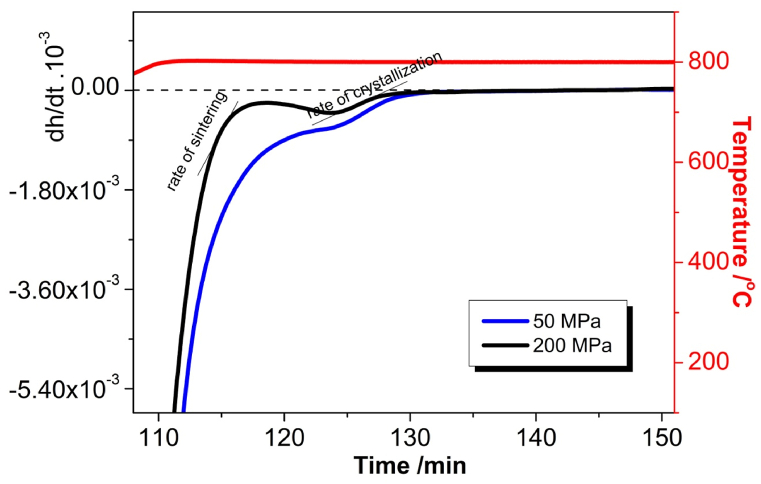
First derivative ODLT curves of sintered press powders.

For the sake of more detailed examination of the both conquering processes (i.e. sintering and crystallization) we used here the first derivative curves with time of the shrinkage curves (i.e. plotting the rates of shrinkage) as shown in Fig. 6. When the differential representation is plotted against the time, this means that the slopes of the first derivative plots correspond to rate of shrink in the resulting differential plots (i.e. here it is visible the change of rate of two separated processes).

Here again the curves more or less are separated into two typical groups of stacking but we plot two representatives of both stacks: the 50 and 200 MPa curves (in Fig. 6). The most important feature of first derivative curves in Fig. 6 is that the inflexion points in the original curves appear here as extremal points (i.e. minima and maxima) in the obtained differential representation.

We may assume that, while the process of sintering in the 50 MPa sample is still not complete, a second shrinkage process initiates, as the crystal phase is more dense compared to the corresponding amorphous phase. This is related to a process of crystallization (phase formation) and connected to a smaller than the sintering shrinkage of the volume of the investigated samples.

In the 200 MPa curve in Fig. 6, both processes of sintering and crystallization are well distinguished, while in the 50 MPa curve, both processes are to some extent overlapped (masked) because of the end of the sintering and the beginning of the phase formation process. At approx. 115 min. laboratory time initiates a process of crystallization, related to crystallization shrinkage. In both curves at the time of 130 min laboratory time, the shrinkage breaks in the same moment for both samples, so that the subsequent crystallization leads to crystallization induced porosity, named CIPs [11].

**Fig. 7:**
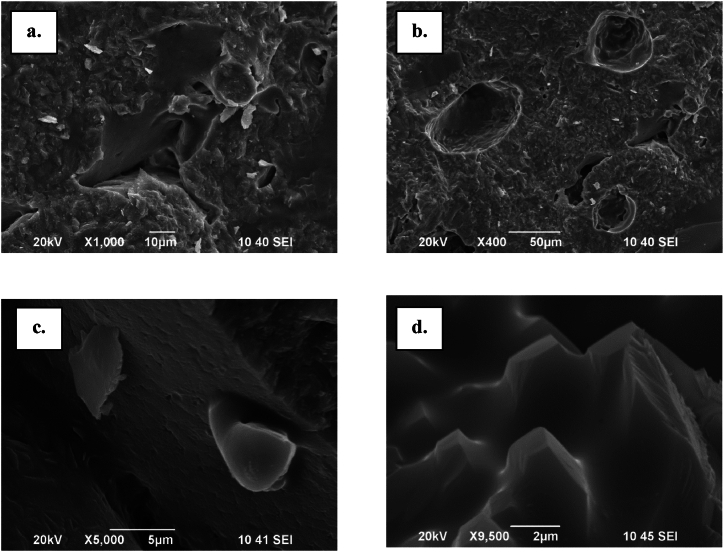
SEM images of surface and fracture of a GB-19 sample: a., b. fracture; c. and d. fine crystalline structure inside a pore.

In Fig. 7a and b are presented respectively SEM photographs from fractures of the studied model glass with the 100 MPa sample. In Fig. 7a and b one can unambiguously distinguish between the two types of pores, namely edgy intergranular porosity and smoother CIPs (crystallizationally induced pores). Fig. 7a possesses smooth irregular shape, while Fig. 7b has semispherical morphology with rough surface. In Fig. 7c and d are shown magnified the inner walls of the pores [10].

**Fig. 8:**
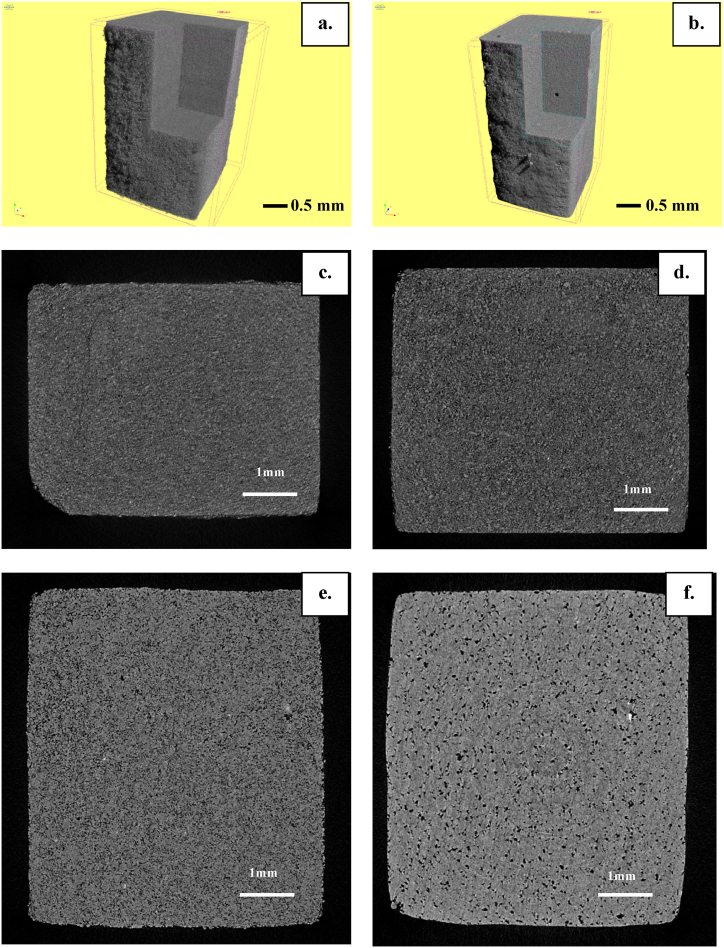
μCT images of glass-ceramic samples obtained with 50 MPa (a, c, e) and 200 MPa (b, d, f) pressing, before and after heat treatment. **a.** and **b.**: 3D reconstruction of the volumes; **c**, **d**: before sintering; **e**, **f**: after sintering.

In Fig. 8 are presented μCT images of the studied glass-ceramics. In Fig. 8a and b are given 3D reconstructions of the volumes of the sintered samples obtained at pressing with 50 and 200 MPa respectively. In Fig. 8c, d, 8e and 8f are shown 2D intersections of both samples before and after sintering heat-treatment.

In both samples the porosity is mainly of closed and spherical type as observed on a micro level with the CT apparatus, with present few coalesced macro pores distributed in the volume [15,16]. The sintering of the particles in both samples is satisfactory and good.

However, the sintering of the 200 MPa sample is to a certain extent better and the surface is much smoother compared to the 50 MPa sample. The closed cells in the 50 MPa sample are on average smaller compared to the 200 MPa sample and its surface is with a rougher morphology.

## 4 Conclusions

In reported research we have demonstrated that the densification of powder samples obtained with different pressure is accomplished within the same time. As a result of the applied various initial pressure, the obtained sintered glass-ceramics possess different final morphology. Different initial porosities lead to obtaining of materials with different end morphologies.

These result highlight the principle of the possibility of obtaining materials with diverse properties by means of variations of the initial pressing force.

The available option of controlling the open porosity could, for instance allow the design of materials used for filtering or catalysis.

## CRediT authorship contribution statement

**N.B. Jordanov:** Writing – original draft. **D. Tatchev:** Investigation, Data curation. **E. Karamanova:** Validation, Data curation. **A. Karamanov:** Writing – original draft, Supervision, Conceptualization.

## Declaration of competing interest

The authors declare that they have no known competing financial interests or personal relationships that could have appeared to influence the work reported in this paper.

## References

[1] Strnad Z. (1986).

[2] Goel A., Ferrari A.M., Kansal I., Pascual M.J., Barbieri L., Bondioli F., Lancellotti I., Ribeiro M.J., Ferreira J.M.F. (2009). Sintering and crystallization behavior of CaMgSi2O6–NaFeSi2O6 based glass-ceramics. J. Appl. Phys..

[3] Rawlings R.D., Wu J.P., Boccaccini A.R. (2006). Glass-ceramics: their production from wastes–a review. J. Mater. Sci..

[4] Monich P.R., Vollprecht D., Bernardo E. (2019). Dense glass-ceramics by fast sinter-crystallization of mixtures of waste-derived glasses. Int. J. Appl. Ceram. Technol..

[5] Chen C., Lan G., Tuan W. (2000). Microstructural evolution of mullite during the sintering of kaolin powder compacts. Ceram. Int..

[6] Lu J., Lu Z., Peng C., Li X., Jiang H. (2014). Influence of particle size on sinterability, crystallisation kinetics and flexural strength of wollastonite glass‐ ceramics from waste glass and fly ash. Mater. Chem. Phys..

[7] Cetin S. (2023). Production of sintered glass-ceramic composites from low-cost materials. Ceram. Int..

[8] Hou Y., Zhang G.-H., Chou K.-C. (2021). Comparison of hot pressing sintering and conventional powder-sintering in preparation of CaO-Al2O3-SiO2-Fe3O4-R2O glass ceramics. J. Non-Cryst. Solids.

[9] Vakifahmetoglu C., Semerci T., Soraru G.D. (2020). Closed porosity ceramics and glasses. J. Am. Ceram. Soc..

[10] Karamanov A., Pelino M. (2008). Induced crystallization porosity and properties of sintered diopside and wollastonite glass-ceramics. J. Eur. Ceram. Soc..

[11] Fokin V.M., Karamanov A., Abyzov A.S., Schmelzer J.W.P., Zanotto E.D., Schmelzer J.W.P. (2014). Glass-Selected Properties and Crystallization.

[12] Bethanis S., Cheeseman C.R., Sollars C.J. (2002). Properties and microstructure of sintered incinerator bottom ash. Ceram. Int..

[13] Karamanov A., Avramov I., Arrizza L., Pascova R., Gutzow I. (2012). Variation of Avrami parameter during non-isothermal surface crystallization of glass powders with different sizes. J. Non-Cryst. Solids.

[14] Karamanov A., Pelino M. (1999). Evaluation of the degree of crystallisation in glass-ceramics by density measurements. J. Eur. Cer. Soc..

[15] Elmoutaouakkil A., Salvo L., Maire E., Peix G. (2020). 2D and 3D characterization of metal foams using X-ray tomography. Adv. Eng. Mater..

[16] Stock S.R. (2008). Recent advances in X-ray microtomography applied to materials. Int. Mater. Rev..

